# Effects of the Salt Stress Duration and Intensity on Developmental and Physiological Features of the Moss *Polytrichum formosum*

**DOI:** 10.3390/plants13111438

**Published:** 2024-05-22

**Authors:** Marija V. Rajčić, Helena Šircelj, Nikolina A. Matić, Sara D. Pavkov, Silvia Poponessi, Tomislav B. Tosti, Aneta D. Sabovljević, Marko S. Sabovljević, Milorad M. Vujičić

**Affiliations:** 1 Institute of Botany and Botanical Garden, Faculty of Biology, University of Belgrade, Takovska 43, RS-11000 Belgrade, Serbia; nikolina.matic@bio.bg.ac.rs (N.A.M.); aneta@bio.bg.ac.rs (A.D.S.); marko@bio.bg.ac.rs (M.S.S.); milorad@bio.bg.ac.rs (M.M.V.); 2 Agronomy Department, Biotechnical Faculty, University of Ljubljana, Jamnikarjeva 101, SI-1000 Ljubljana, Slovenia; 3 Department of Biology and Ecology, Faculty of Sciences, University of Novi Sad, Trg Dositeja Obradovića 3, RS-21000 Novi Sad, Serbia; 4 Department of Life and Environmental Sciences, Botany Section, University of Cagliari, IT-09123 Cagliari, Italy; 5 Faculty of Chemistry, University of BelgradSe, Studentski trg 12–16, RS-11158 Belgrade, Serbia; tosti@chem.bg.ac.rs; 6 Center of Plant Biotechnology and Conservation (CPBC), Takovska 43, RS-11000 Belgrade, Serbia; 7 Department of Botany, Institute of Biology and Ecology, Faculty of Science, Pavol Jozef Šafárik University in Kosice, Mánesova 23, SK-040 01 Košice, Slovakia

**Keywords:** bryophyte, NaCl, hair-cap moss, stress tolerance, resistance, biochemical characteristics, growth, genotypes

## Abstract

The two accessions of the polytrichaceous moss species *Polytrichum formosum*, namely German and Serbian genotypes, were subjected to salt stress, aiming to study the species’ developmental and physiological features. Various concentrations of sodium chloride were applied to an axenic *in vitro* culture of the two moss genotypes, and the growth parameters as well as physiological feature changes were followed. As inferred by the morpho-developmental parameters and survival index, the Serbian genotype showed higher resistance to salt stress as compared to the German one. However, both moss genotypes survived the highest applied concentration (500 mM). As expected, short exposures to salt were rather easily overcome. No clear patterns in sugar content and changes were observed during the stress, but they are surely included in salt stress response and tolerance in *P. formosum*. Longer stress increased total chlorophyll content in both genotypes. In short-term applied salt stress, the Serbian genotype had a higher total chlorophyll concentration to control unstressed plants, while the German genotype decreased the total amount of chlorophyll. Similarly, carotenoids were shown to be significantly higher in the Serbian genotype, both in unstressed and treated plants, compared to the German one. The contents of tocopherols were higher in the Serbian genotype in controlled unstressed and subsequently short- and long-stressed plantlets compared to the German accession. In general, we can assume that *P. formosum* is unexpectedly tolerant to salt stress and that there are differences within various accessions of overall European populations, as referred by two randomly selected genotypes, which is most probably a consequence of different genetic structure.

## 1. Introduction

Bryophytes, a large group of plants with over 20,000 named species (e.g., [1]), inhabit almost all habitats on the Earth except the seas. Their ancestors were among the first plants to settle upon the harsh land environment [2]. However, some species can be found in brackish waters as well as in salty areas, and these are usually considered as bryo-halophytes, i.e., halo-bryophytes due to their peculiar life strategies and requirements [3].

Bryo-halophytes are exclusively recorded in salt substrate or brackish water in nature. Nevertheless, some experimental approaches in controlled conditions have shown some of them to be facultative halophytes (e.g., *Enthostodon hungaricus* (Boros) Loeske) and the low competitiveness with other bryophytes and biochemical, developmental, and physiological features seem to push them to such places rather than salt dependence [4]. The others indeed require salts for the normal growth (e.g., *Riella helicophylla* (Bory et Mont.) Mont. [5]. Some species, never seen in salty environments in nature, also show surprising tolerance to salt (please refer to [6,7] for more details), implying the existence of a genetic memory inherited from ancestors [8] and expressed in certain circumstances, a joint response pathway to some other stress, or both. Indeed, for the model and non-halophytic moss, namely *Physcomitrium (Physcomitrella) patens* (Hedw.) Mitt., it is shown to tolerate a high amount of sodium chloride [8]. The same authors [8] assumed that there is a certain molecular memory related to ancestors which were among the first plants that left the sea.

Mosses, on the other hand, have many advantages to be studied in a fundamental approach, since they are haploid organisms and rather easy and cheap to handle. However, they are often avoided in studies, even though they are the second largest group of terrestrial plants, due to their minute size, small yield and tiny biomass, high sensitivity to small environmental changes, and problems in sampling of axenic material and identifications. However, they can offer many responses and trial biotechnological solutions that can be applied in crop production, plant growth and development, but also in healing environmental disorders.

Land salinization is one such problem in the modern era that highly affects nature and civilization. Thus, we address this study to moss relation to salt stress. With an aim to test whether the non-halophytic moss *Polytrichum formosum* (Hedw.) G. L. Smith can tolerate salt stress, we studied its developmental and physiological features. Moreover, we studied two distant populations, further referred to as genotypes. The salt stress was simulated in an *in vitro* culture of these two populations established axenically for these purposes. The aim of this study was to determine how selected *P. formosum* genotypes react and recover from short- and long-term stress induced by NaCl, by examining survival, multiplication index, chlorophyll, carotene, xanthophyll, and tocopherol content, and sugar profile and content.

## 2. Results

### 2.1. In Vitro Culturing

The cultures of two genotypes of *P. formosum*, namely German and Serbian accessions, were obtained from spores. The sterilized spores were germinated after 7 days. The pieces of primary protonemata were then transferred to various media types to get fully developed gametophores which were the subject of further studies. The best biomass achievements were at MS half strength medium, as compared to BCD and KNOP media types. At the same medium type both genotypes produced buds 17 days on average after germination and the gametophores (small plantlets) were visible within 30 days. They continued to grow to fully developed plants that were similar to the native ones and to make cushions up to 7–9 cm in diameter within 90 days. The tips of these plants were used for subsequent propagation, and they were able to develop secondary protonemata and new stems.

### 2.2. Morphogenesis

Both studied genotypes of *P. formosum* did survive all treatments applied (10–500 mM) both in short-term NaCl exposure (4 days at salt enriched MS/2 medium and 24 days in recovery medium with no salt added) and long-term exposure (28 days constantly at MS/2 medium enriched with NaCl). As expected, survival rates decreased with the salt exposure time and concentration increase (Table 1). The survival rate of the Serbian genotype is shown to be slightly higher. Both moss genotypes expressed resistance and unexpected tolerance to stress caused by NaCl up to rather high 500 mM with 50% survival rate (Table 1), both in short- and long-term salt stress.

The development of visible secondary protonemata was not documented in any treatment, but it was also not visible in any of the tested accessions in the control group during 28 days.

**Figure 1:**
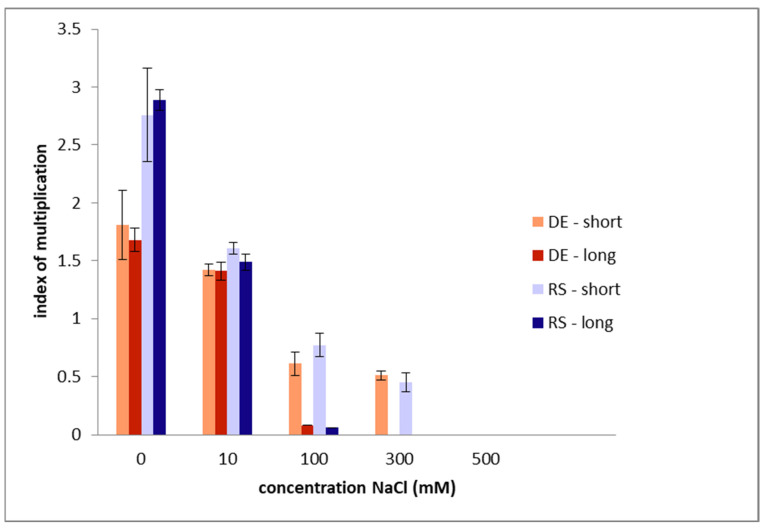
Index of multiplication (records of new shoots development after 28 days) in two salt- stressed genotypes of *Polytrichum formosum* in various concentrations of NaCl applied for short- (4 days) and long-term (28 days) stress. RS—Serbian and DE—German genotypes. Data are presented as mean values ± standard errors.

Nevertheless, newly developed shoots were counted at the end of treatments, i.e., on 28th day (Figure 1). As compared to control moss groups, the salt-stressed plants decreased in new shoot development. In long-term exposure, new shoots stopped development at 300 mM NaCl, and their index of multiplication was rather similar and low in both tested geophytes. As referred from the index of multiplication of short-term exposure and recovery up to 28 days, the development of new shoots linearly decreased until 300 mM and was not seen at 500 mM in either genotypes.

### 2.3. Pigments

**Figure 2:**
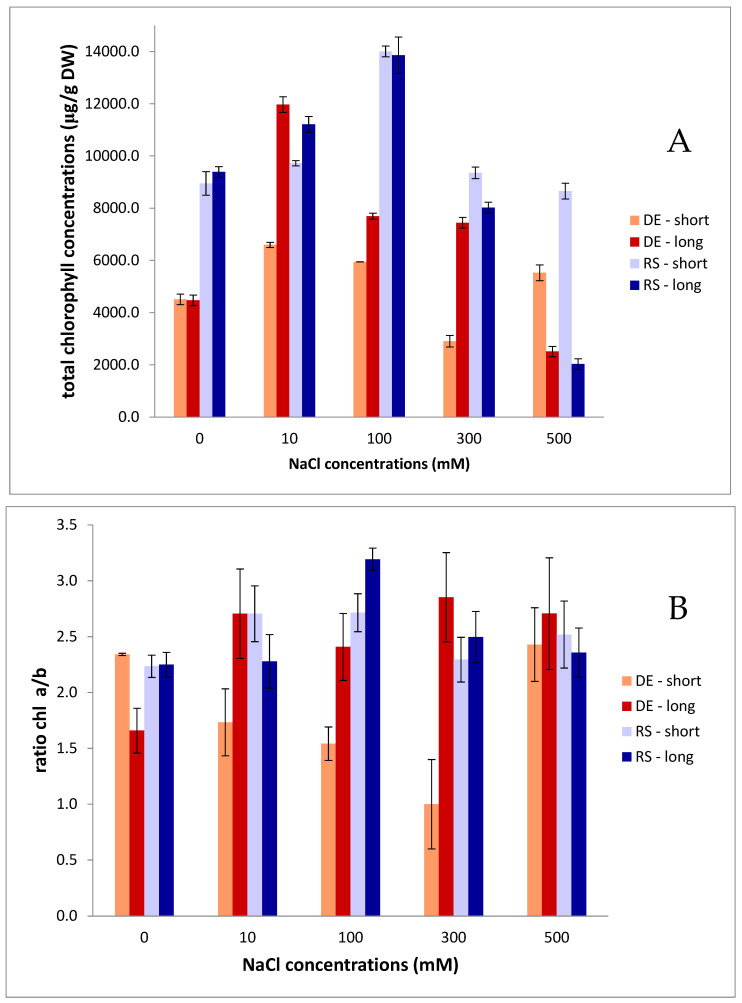
Total chlorophyll content (**A**) and chlorophyll a/b ratio (**B**) in two accessions of *Polytrichum formosum* under salt stress (DE—German accession; RS—Serbian accession; long—long-term salt exposure (24 days); short—short-term salt exposure (4 days)). Data are presented as mean values ± standard errors.

The tested accessions (gametophores) exhibited a rise in total chlorophyll content with the increase of salt concentration in long-term exposure up to 300 mM salt in medium, but they were significantly lower at 500 mM. However, in short-term exposure (4 days) to salt, with salt content in the media increasing, total chlorophylls increased for the Serbian genotype, but decreased in the German genotype (Figure 2). The chlorophyll a/b ratios obtained with the two genotypes appear to be evenly distributed. However, the Serbian genotype seems to be more sensitive to salt stress as compared to the control population chlorophyll a/b ratio, while German accession showed tolerance to some extent (Figure 2). The stress differences between the two genotypes were best visible at concentrations of 10, 100 and 300 mM.

The amounts of total carotenoids were different in two *P. formosum* genotypes, although they have similar patterns under treatments. The more resistant Serbian genotype had significantly higher levels of carotenoids both in the control group of plants and plants under treatments compared to German accession.

**Figure 3:**
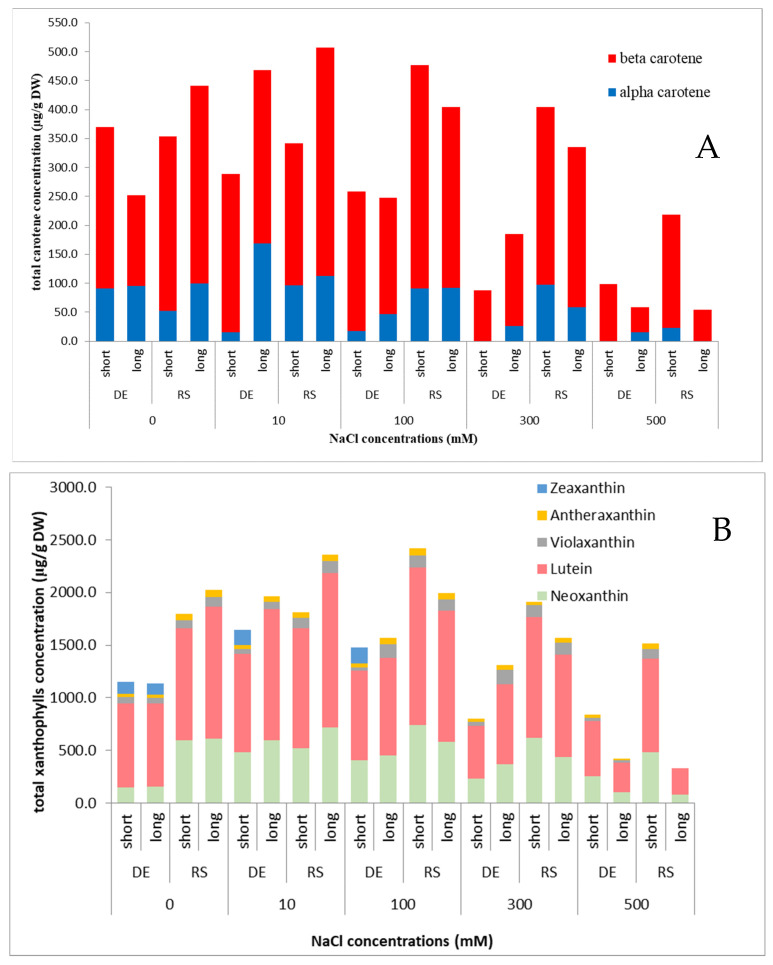
Total carotenes (**A**) and xanthophylls (**B**) and their type contents in two accessions of *Polytrichum formosum* under salt stress (DE—German accession; RS—Serbian accession; long—long-term salt exposure (24 days); short—short-term salt exposure (4 days)).

Total carotenes remained rather high in the Serbian accession, both in short- and long-term exposure to various concentrations of salt, in contrast to the German accession where it decreased in short-term stress nearly linearly, and increase in long-term stress only in the low salt concentration (10 mM) and then rapidly decreased with rising salt content in the medium (Figure 3). It has to be noted that α carotene was not detected in the Serbian accession at long-term exposure 500 mM, while in the German accession it was absent in 300 and 500 mM NaCl-enriched media at short-term treatments.

Total xanthophyll amount showed similar patterns in both tested genotypes, which at first rise with the salt concentrations increase up to 100 mM and then significantly decrease. However, zeaxanthin was not detected in one of the genotypes (Serbian) and in the German genotype, it was detected only at lower concentration of applied NaCl. Figure 3 clearly shows vitality loss when 500 mM were applied, although but the Serbian genotype can survive better than the German accession in short exposure to salt stress.

### 2.4. Tocopherols

**Figure 4:**
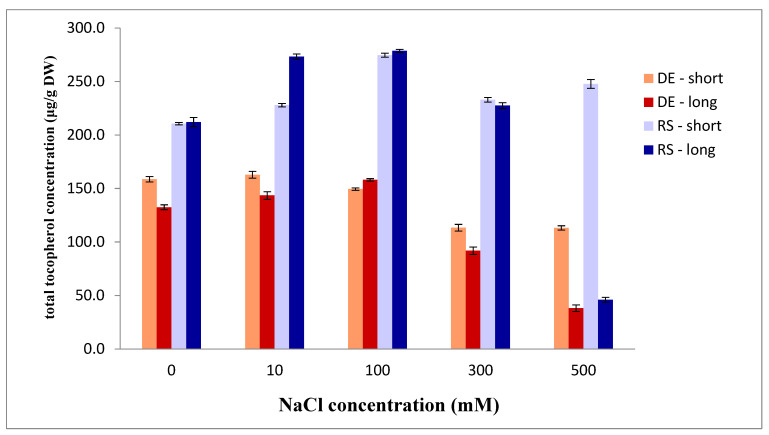
Total amounts of tocopherols in two accessions of *Polytrichum formosum* under salt stress (DE—German accession; RS—Serbian accession; long—long-term salt exposure (24 days); short—short-term salt exposure (4 days)). Data are presented as mean values ± standard errors.

The total tocopherols and their species in the moss *P. formosum* clearly show that the higher amount is present in all tested and control groups of the Serbian accession compared to the German one (Figure 4). The difference between accessions in short-term salt stress is higher with the increase of salt concentrations. However, in long-term salt stress the pattern of total tocopherols is similarly increased until the NaCl amount up to 100 mM, and then decreased until 500 mM, where it is almost equal among two genotypes. It has to be noted that the δ-tocopherol was not detected in total amount in any accessions or treatments, and that γ-tocopherol is rarely detected and in trace. Thus, the amount of total tocopherols is mainly α-tocopherol. Bearing in mind that it has high antioxidant activity, it is not surprising that in lower NaCl concentration and shorter stress exposure its level is high and that 500 mM NaCl is limiting one for the survival of this species (in both accessions) in long-term exposure.

### 2.5. Sugars

The sugars seem to play an important role in maintenance of moss organisms under various stresses, osmotic, salt or drought as shown previously [9,10,11]. The sugar evidence under various developmental stages and environmental conditions in mosses is a first step in elucidation of their involvements in various processes in moss organisms. However, the role of specific sugars remains obscure, whether functional and/or structural, and needs further elucidations.

The sugars detected during treatments and within control groups include 22 different sugars and sugar alcohols, namely: glucose, fructose, melibiose, sucrose, turanose, maltose, sorbitol, arabinose, trehalose, glycerol, galactose, galactitol, ribose, isomaltose, isomaltotriose, maltotriose, mannitol, xylose, panose, rhamnose, raffinose and strachyose.

All measured sugars express different patterns per treatments (salt concentration and exposure variation) and between tested moss accessions. The results obtained clearly demonstrate they varied very much (Table 2). In the German accession, glucose in short-term exposure decreased with salt concentration increase while in long-term exposure the same concentration resulted in a reversed pattern so that the glucose amount was significantly higher in plants grown at the highest 500 mM sodium chloride for 28 days. Fructose was in lower concentrations compared to glucose but showed similar reversed pattern as referred to exposure period. Sucrose varied with no clear pattern in tested genotypes or treatments. In the Serbian accession, glucose showed a completely opposite pattern compared the German accession. Fructose in both short- and long-term stress treatments showed slight increase except at highest salt concentration in the long-term experiments. Turanose seems to be significantly represented in both accessions and to vary with salt stress, but no clear pattern was detected.

## 3. Discussion

The presence of carotenes, especially β-carotene, in higher amounts, imply its role in oxidative stress regulation [12]. The smaller portion of α-carotene is permanent in tested moss samples under all treatments and even absent under the highest NaCl concentration stress suggesting the higher importance of β-carotenes in salt stress tolerance. However, its role can be blurred but significant in short-term salt stress and high salt concentration in *P. formosum*, and the α/β carotene ratio seems to be rather characteristic in optimal, i.e., unstressed conditions and can be regarded as a marker for salt stress tolerance/resistance, i.e., suboptimal growth conditions.

The α-carotene absence in higher concentrations of salt (300 and 500 mM) in both moss accessions and long- and short-term treatments suggests its importance and secondary role in mechanisms of salt stress tolerance in plants [13], as well as in *P. formosum*. Carotene content is rather related with environmental light conditions, but in these cases, all plants in treatments were exposed to control light intensity and duration in growth chambers and thus this is no explanation for its absence. It can be assumed, that it participates in some other mechanisms as well.

Zeaxanthin seems to be among the first xanthophyllsto be included into antioxidative stress response [14]. In higher salt concentrations and long-term exposure to salts stress, zeaxanthin was not present significantly in these mosses (Figure 3), suggesting its pivotal role in salt tolerance compared to all tested xanthophylls in *P. formosum*. The concentration of zeaxanthin decreases. The reasons for decrease of zeaxanthin can be regarded as its degradation or blocked synthesis or most likely conversion to violaxanthin or antheraxanthin [14]. It seems that salt stress does not imply any significant changes of other xanthophyll species detected in *P. formosum*.

Tocopherol acts as an antioxidant and prevents lipid peroxidation in membranes [15]. The increase of α-tocopherol favors stress tolerance and decreased levels of oxidative damage. In rather better adapted organisms to salt stress, it is highly present during the stress, and so this also confirms the better adaptation of the Serbian origin accession of *P. formosum* to salt stress compared to the German one. Since tocopherol content is known to increase with age of the leaf and also with slowly developing stress, and decreases under severe stress [16], we can infer that high NaCl concentration is a rather huge stress for both studied genotypes of *P. formosum*.

Sucrose, fructose, and glucose were the most dominant sugars, similar to that found in the native population of Serbian accession, where they varied significantly among the seasons [17]. Similarily as in experimental plants, trehalose and turanose were marked during drought and cold stress suggesting some role of these sugars as response to stresses and suboptimal growth conditions [18]. Thus, similar reaction pathways to salt, drought and cold stress are most probably present in the moss *P. formosum*. Some of the sugars firstly ever reported as permanent constituents in *P. formosum* in native populations are also documented in laboratory plants of both genotypes regardless of stress applied. The quantitative changes of sugars demonstrate carbohydrate dynamics in relation to environmental changes wheather experimental or natural ones, and thus are probably physiologically significant compounds among *P. formosum* chemical constituents, yet with no clear patterns. They can be signalling, energy storage as well as carbon supplier molecules. We can assume that under NaCl stress, amount of glucose, fructose and sucrose are detected as slightly higher compared to control plants, due to changed (decreased or blocked) sugar transport in plantlets.

## 4. Material and Methods

### 4.1. Plant Material

The spores were taken from the BEOU-Bryo collection (Belgrade University Bryophyte Collections) where the vouchers are kept. Capsule on a mature sporophyte selected from each of the two accessions: German one (BEOU-Bryo 00506, Konigsforst Bei Köln, Cologne, Germany; 14 February 2006; leg./det. Aneta Sabovljević and Marko Sabovljević) and Serbian one (BEOU-Bryo 03467, Rakovac, Fruška Gora National Park, Serbia; 18 March 2001; leg./det. Aneta Sabovljević and Marko Sabovljević).

*In vitro* cultures of the moss *Polytrichum formosum*, two genotypes (Serbian and German), were established as described in [19]. The sterile spores were released to agarose gel for germination. The pieces of germinated protonemata were transfered to three different media types (namely MS, BCD and KNOP) to achieve quick and fully developed gametophores. Observation method was used to test if the moss is similar to the natural ones. Leafy gametophores were observed to test vitality and used in stress treatments and tests as well as in biochemical analyses. Additionally, the number of newly developed shoots and patch diameter were the characters to select medium for further stress tests. For the media salt content, please refer to [20,21,22]. Thus, once the gametophore achieved, it was used as a source for all further sub-cultures and all the material is originated from the single spore/genotype for each of the accessions.

### 4.2. Experimental Design

Axenic *in vitro* cultures of the two different *P. formosum* genotypes were established and the full development of gametophores was achieved. The selected moss gametophores were grown on solid MS half-strength medium [20] with no sucrose added, to achieve quick growth and well-developed plants prior to the experimental tests. The pH of the media was adjusted to 5.8 before autoclaving at 121 °C for 30 min.

Salt stress expreriments were conducted on MS half strength solid medium supplemented with different NaCl concentrations before autoclaving (10, 100, 300, and 500 mM NaCl). In experiment type I, the mosses were grown on MS half-strength medium containing NaCl for 4 days, which simulated short-term stress, and afterward these plantlets were transfered to NaCl-free medium up to 28 days. Experiment type II represented long-term stress, where the mosses were grown on MS half-strength medium containing NaCl for 28 days. The control plants were grown on MS half-strength salt-free medium.

The culture conditions were set at 18 ± 2 °C, under a long-day photoperiod (16 h/8 h light/dark). All the plants tested here were grown under the same controlled laboratory conditions including the light quality and intensity (47 µmol m^−2^ s^−1^ irradiance). After 28 days the plant material was collected and frozen at −70 °C until further analysis.

### 4.3. Analysis of Chloroplast Pigments

Pigments (chlorophyll -a, -b, α-carotene, β-carotene, violaxanthin, antheraxanthin, neoxanthin, zeaxanthin and lutein) were analyzed following methodology described previously [23]. Plant material (100 mg of dry and frozen gametophores) was powdered. Pigments were isolated with 5 mL of ice-cold acetone, using T-25 Ultra-Turrax (Ika-Labortechnik, Staufen im Breisgau, Germany) homogenizer for 25 s. All extraction procedures and HPLC analyses were performed according to previously applied protocol [24]. Compound identification was done by addition of standards and comparing the spectra and the retention times.

### 4.4. Tocopherols

Tocopherols (a-, c-, d-tocopherol) were isolated and identified as reported previously [23]. The concentrations of tocopherols were calculated using corresponding external standards.

### 4.5. Sugars

Sugars (glucose, sucrose, fructose, maltose, and trehalose) were isolated, analyzed and identified following methodology as described in [17]. The concentrations of different sugars were calculated using standards: glucose, sucrose, fructose, maltose, trehalose, galactose, raffinose and maltotriose (the Tokyo Chemical Industry, TCI, Europe, Zwijndrecht, Belgium or Tokyo, Japan); sorbitol, manose, galactitol, rhamnose and mannitol (Sigma-Aldrich, Steinheim, Germany).

### 4.6. Statistics

In this study, descriptive statistics were exported from the Excel Program. All data are presented as mean values ± standard errors where applicable. The replicates were at least 40 per test, i.e., 10 petri plates with 4 explants, and 3 technical repetitions on 3 samples were used for all measurements.

## 5. Conclusions

The results obtained clearly showed that the polytrichaceous moss *P. formosum* can tolerate salt (NaCl) stress to some extent and that various biochemical and physiological features are affected by stress. The two compared genotypes are expressing different reaction pathways and the Serbian genotype seems to be slightly more resistant to salt stress. These facts led to the conclusion that genetic constitution within the same species plays a pivotal role in species adaptive ecology and targeted niche survival, i.e., certain environmental factor amplitude reaction. Additionally, it seems to be that molecular memory to salt resistance is still present within the genome of this non-halophytic moss biological entity in accordance with distant ancestors. These will be the subject of further testing different genotypes (e.g., those growing in more salty conditions compared to those growing in salt-free environment) and/or by studying *in vitro* genotypes sub-cultivated for many generations in one condition and then transferred to a stressed one. Selected gene up-regulation and down-regulation, i.e., expressions can also lead to a conclusion of stress molecular memory in well-designed tests in control laboratory conditions. The results presented here are pioneer and preliminary in the field of salt stress in non-halophytic bryophytes and are therefore significant for further tests, considerations, and elucidations on salt stress in mosses and allies.

## Figures and Tables

**Table 1:** Survival rate (%) of tested accessions of *Polytrichum formosum* in short-term (4 days) and long-term (24 days) salt stress.

Survival (%)	German Genotype		Serbian Genotype	
NaCl Concentration (mM)	Short-Term Stress	Long-Term Stress	Short-Term Stress	Long-Term Stress
0	100 ± 0.00	100 ± 0.00	100 ± 0.00	100 ± 0.00
10	100 ± 0.00	100 ± 0.00	100 ± 0.00	100 ± 0.00
100	100 ± 0.00	76.67 ± 1.80	100 ± 0.00	100 ± 0.00
300	98.33 ± 0.50	70 ± 2.20	98.33 ± 0.60	88.33 ± 0.50
500	80 ± 0.80	50 ± 0.90	78.33 ± 0.75	73.33 ± 0.80

**Table 2:** Heat maps of sugars and sugar alcohols (mg/mL) variation in two accessions of *Polytrichum formosum* (A—German genotype (DE); B—Serbian genotype (RS)) detected during short- and long-term salt stress.

(A)											
	DE	SHORT-TERM STRESS					LONG-TERM STRESS				
	NaCl (mM)	0	10	100	300	500	0	10	100	300	500
**Monosaccharides**	**Arabinose**	7.910	6.954	8.026	3.981	5.531	6.379	6.951	7.811	9.367	1.567
	**Galactose**	13.481	11.851	13.678	6.785	10.952	10.871	11.000	12.362	7.917	10.263
	**Glucose**	82.542	60.010	48.560	49.938	33.207	65.290	56.371	82.681	90.225	117.227
	**Fructose**	35.600	23.375	16.408	15.597	13.590	27.071	23.771	38.467	45.296	61.979
	**Rhamnose**	0.787	0.657	0.708	0.685	0.680	0.869	0.858	0.850	0.869	0.862
	**Ribose**	5.824	8.380	7.562	5.075	6.496	2.526	3.781	4.396	5.489	4.048
	**Xylose**	4.483	7.530	7.435	7.455	7.484	4.511	4.546	4.531	4.511	4.525
**Disaccharides**	**Sucrose**	42.767	40.912	64.224	37.488	47.080	34.717	58.682	40.146	56.695	71.342
	**Isomaltose**	1.982	0.362	1.651	0.341	1.011	1.486	0.217	0.991	1.284	0.995
	**Maltose**	7.183	4.235	1.232	8.110	6.058	1.203	2.965	4.606	4.666	3.580
	**Melibiose**	2.512	3.263	3.050	1.347	0.639	0.419	0.563	2.535	2.983	7.553
	**Trehalose**	3.080	3.126	6.960	8.515	6.146	8.244	6.820	2.356	5.336	8.189
	**Turanose**	37.404	29.327	40.174	27.207	25.503	24.863	36.927	38.573	38.573	28.834
**Trisaccharides**	**Isomaltotriose**	0.701	0.779	0.596	0.608	0.645	0.487	0.818	1.920	0.541	0.942
	**Maltotriose**	0.884	2.134	1.151	0.925	1.241	1.390	1.174	1.639	0.756	1.240
	**Panose**	1.065	0.901	0.899	0.911	0.905	1.742	1.661	1.716	1.742	1.715
	**Raffinose**	1.317	1.151	1.175	1.159	1.152	1.184	1.131	1.177	1.184	1.169
**Tetrasaccharides**	**Stachyose**	0.623	0.558	0.598	0.573	0.565	0.665	0.652	0.635	0.665	0.654
**Sugar alcohols**	**Galactitol**	0.910	2.977	0.141	0.375	1.038	1.279	1.960	3.475	0.193	1.477
	**Glycerol**	4.362	7.589	4.203	4.204	7.044	5.813	18.982	3.531	4.080	8.101
	**Mannitol**	1.437	1.247	1.229	1.304	1.251	1.480	1.548	1.354	1.480	1.466
	**Sorbitol**	12.595	12.760	13.198	13.648	14.586	11.051	14.981	12.225	12.346	12.051
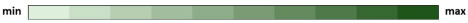											
(**B**)											
	**RS**	**SHORT-TERM STRESS**					**LONG-TERM STRESS**				
	**NaCl (mM)**	**0**	**10**	**100**	**300**	**500**	**0**	**10**	**100**	**300**	**500**
**Monosaccharides**	**Arabinose**	6.455	3.940	8.035	2.184	9.188	6.849	5.403	8.660	6.649	0.300
	**Galactose**	12.377	6.715	13.693	12.847	15.431	12.909	9.208	15.989	5.116	15.418
	**Glucose**	72.779	31.111	64.167	74.465	91.944	58.276	91.636	65.023	58.054	31.319
	**Fructose**	21.786	29.936	24.995	30.416	32.004	18.714	30.095	36.638	35.830	22.574
	**Rhamnose**	1.973	1.209	1.139	1.167	1.372	2.043	1.183	2.074	2.013	1.828
	**Ribose**	3.437	9.015	2.855	3.188	4.624	2.631	2.453	8.721	1.992	3.949
	**Xylose**	6.195	2.951	3.002	2.971	3.780	6.203	2.947	6.235	6.248	5.409
**Disaccharides**	**Sucrose**	45.591	11.190	78.936	82.235	88.118	43.059	71.888	72.947	26.991	13.824
	**Isomaltose**	1.546	0.230	0.190	0.821	0.697	0.930	0.552	0.271	0.202	0.489
	**Maltose**	2.520	2.370	1.845	2.084	2.146	2.417	3.168	2.319	2.196	1.880
	**Melibiose**	4.231	5.044	3.011	5.051	2.999	3.215	1.986	2.102	0.986	0.542
	**Trehalose**	3.944	6.922	7.926	2.570	12.840	0.958	6.674	8.119	9.293	9.661
	**Turanose**	24.891	12.885	22.058	34.683	24.404	25.843	32.560	26.663	14.593	5.328
**Trisaccharides**	**Isomaltotriose**	0.591	0.572	0.690	2.197	1.012	1.854	0.634	0.651	0.768	0.977
	**Maltotriose**	1.041	0.954	0.820	1.070	0.971	0.953	0.869	1.207	0.944	0.993
	**Panose**	7.118	4.887	4.993	4.808	5.451	3.588	4.859	3.608	3.619	3.316
	**Raffinose**	1.278	1.237	1.234	1.266	1.254	1.304	1.258	1.319	1.268	1.288
**Tetrasaccharides**	**Stachyose**	0.871	0.737	0.777	0.789	0.794	0.806	0.719	0.827	0.813	0.791
**Sugar alcohols**	**Galactitol**	0.619	0.701	0.907	2.117	0.724	1.591	0.721	2.035	2.269	2.654
	**Glycerol**	4.335	13.385	13.514	3.444	8.670	2.255	3.837	4.570	8.026	8.257
	**Mannitol**	2.258	1.515	1.440	1.520	1.683	1.961	1.456	2.170	2.154	6.830
	**Sorbitol**	12.691	12.376	8.289	9.097	10.613	7.396	13.028	13.135	13.697	11.939
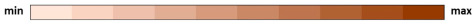											

## Data Availability

Data are contained within the article.
